# Achieving Complete Cytoreduction After Systemic Therapy for Stage IV Renal Carcinoma With Multicompartment Recurrence and Peritoneal Carcinomatosis: A Case Report

**DOI:** 10.1155/crom/9036511

**Published:** 2026-08-27

**Authors:** Juan Pablo Usubillaga, Rodolfo Varela, Yeison Reina, Diana Carolina Franco, Juan Sebastián Valderrama, Martín Zapata-Laguado, Daniel Suso-Palau

**Affiliations:** ^1^ Department of Urologic Oncology, Instituto Nacional de Cancerología, Bogotá, Colombia, incan-mexico.org; ^2^ Department of Clinical Oncology, Instituto Nacional de Cancerología, Bogotá, Colombia, incan-mexico.org

**Keywords:** cytoreductive surgery, immunotherapy, metastatic, peritoneal metastasis, renal cell carcinoma

## Abstract

Clear cell renal cell carcinoma (ccRCC) most commonly metastasizes to the lungs, bones, liver, and brain, whereas peritoneal involvement is exceedingly rare and typically associated with aggressive disease and poor prognosis. Although the combination of immune checkpoint inhibitors and tyrosine kinase inhibitors has significantly improved outcomes in metastatic ccRCC, reports documenting substantial pathological responses following these regimens remain limited. We describe the case of a 31‐year‐old male initially diagnosed with localized ccRCC (pT3aN0M0) after presenting with spontaneous retroperitoneal hemorrhage and undergoing radical nephrectomy. Seven months later, he developed progressive ascites and was restaged with extensive multicompartment recurrence, including peritoneal metastases, hepatic lesions, retroperitoneal lymphadenopathy, and tumor thrombus in the infrahepatic inferior vena cava. The patient was classified as poor risk according to the International Metastatic RCC Database Consortium criteria. Systemic therapy with pembrolizumab and lenvatinib was initiated, resulting in greater than 50% radiologic tumor reduction and marked clinical improvement after 14 cycles. Following this favorable response, complete cytoreductive surgery targeting all affected compartments was performed. Histopathological examination revealed extensive tumor necrosis with less than 1% residual viable tumor, confined to a single hepatic segment. The patient remains asymptomatic under close surveillance. To our knowledge, this is the first reported case of ccRCC with multicompartment metastatic disease involving peritoneal carcinomatosis treated with pembrolizumab plus lenvatinib followed by complete cytoreductive surgery, achieving near‐complete pathological response, highlighting the potential of multimodal strategies in selected patients with advanced disease.

## 1. Introduction

Clear cell renal cell carcinoma (ccRCC) is the most frequent subtype of adult renal cancers, accounting for 70%–80% of diagnoses [1]. Although metastases are commonly observed in the lungs, bones, liver, and brain, peritoneal involvement is notably infrequent, with a reported incidence of less than 1% in both clinical and autopsy series [2, 3]. When present, peritoneal metastases are generally associated with advanced disease and unfavorable prognosis [4]. This presentation, however, rarely occurs in isolation, typically coexisting with multicompartmental hematogenous or lymphatic dissemination that poses an exceedingly challenging therapeutic scenario.

Currently, the combination of immune checkpoint inhibitors and tyrosine kinase inhibitors has significantly transformed the prognosis for patients with metastatic ccRCC. Evidence from trials such as CLEAR has demonstrated objective response rates exceeding 70%, with up to 20% complete responses based on imaging criteria [5]. However, the literature predominantly focuses on radiological outcomes, and reports documenting significant pathological tumor regression after systemic therapy are limited.

We present the case of a young patient with ccRCC and multicompartment recurrence characterized by extensive peritoneal carcinomatosis, hepatic lesions, and retroperitoneal involvement, who was successfully managed with pembrolizumab plus lenvatinib, followed by complete surgical cytoreduction. Pathological analysis revealed extensive tumor necrosis and a minimal proportion (< 1%) of residual viable tumor. This report highlights the potential of systemic therapies to achieve near‐complete responses even in advanced, multicompartmental, and aggressive stages of the disease and strengthens the evidence supporting deferred cytoreductive surgery in selected patients. This case report was prepared in accordance with the CARE (CAse REport) guidelines (Supporting Information (Available here)).

## 2. Case Presentation

In January 2023, a 31‐year‐old male, previously in good health, presented with acute onset of intense left flank pain. An abdominal computed tomography scan revealed a retroperitoneal collection consistent with hematoma, along with a left renal mass suggestive of spontaneous hemorrhage from an underlying tumor. The patient underwent a laparoscopic radical nephrectomy at an outside institution via a transabdominal approach, which was completed without complications. According to the surgical description, the specimen was extracted intact using a Viaflex bag, with no evidence of intraoperative tumor spill during the extraction procedure.

Histopathological examination confirmed ccRCC, WHO/ISUP grade 3, with tumor thrombus involving the renal vein and approximately 10% tumor necrosis. No sarcomatoid or rhabdoid features were observed, and all surgical margins were negative. The disease was staged as pT3aN0M0. Following the primary surgery, the patient did not receive routine surveillance imaging until severe symptoms arose, as he was subsequently referred to our institution.

**Figure 1:**
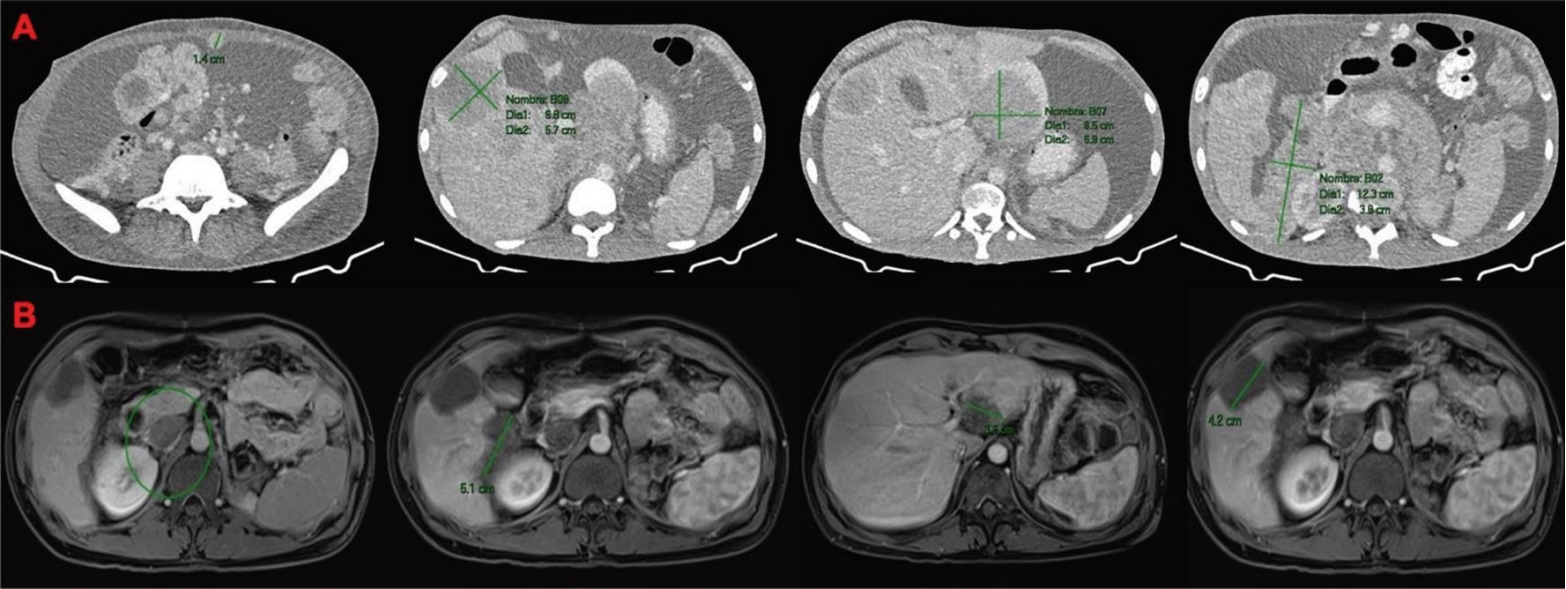
(A) Axial contrast‐enhanced computed tomography (CT) images from April 2024 demonstrate multiple intra‐abdominal metastatic lesions, including peritoneal implants, retroperitoneal lymphadenopathy, and liver metastases. (B) Axial magnetic resonance imaging (MRI) after systemic therapy with pembrolizumab plus lenvatinib shows marked reduction in lesion size across all metastatic sites, suggesting a significant partial response per RECIST v1.1.

Seven months after surgery, the patient presented to our institution with progressive abdominal distension. Upon admission, he was found to have large‐volume ascites that required therapeutic paracentesis. Follow‐up restaging CT imaging at this point showed the massive ascites, multiple peritoneal implants, hepatic lesions consistent with metastases, retroperitoneal lymphadenopathy, and tumor thrombus extending into the infrahepatic inferior vena cava (Figure 1A). No pulmonary involvement was observed. The disease was restaged as metastatic ccRCC (Stage IV, M1), representing a highly aggressive multicompartment recurrence. The patient was classified as poor risk according to the International Metastatic RCC Database Consortium (IMDC) criteria based on clinical and laboratory parameters.

In April 2024, systemic therapy was initiated with pembrolizumab (200 mg IV every 3 weeks) and lenvatinib (20 mg orally daily). By March 2025, after completing 14 cycles, the patient achieved a > 50% reduction in measurable lesions, consistent with a partial response per RECIST v1.1 (Figure 1B). This was accompanied by clinical improvement. The treatment was well tolerated without the need for dose modifications. Reported adverse events included Grade 2 fatigue, Grade 1 diarrhea, and Grade 2 hypertension, which was controlled with losartan and amlodipine.

**Figure 2:**
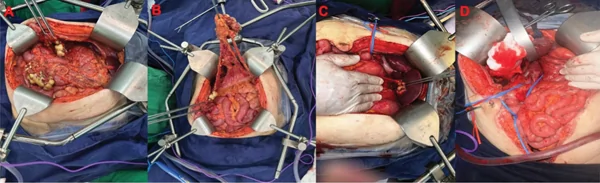
Representative intraoperative photographs taken during cytoreductive surgery. (A) Peritoneal carcinomatosis with tumor implants on the greater omentum, parietal peritoneum, right diaphragmatic dome, pelvis, and spleen. (B) Mobilization and resection of the greater omentum showing extensive tumor involvement. (C) Identification and control of hepatic metastases in Segments II and V. (D) Exposure of the intrahepatic portion of the inferior vena cava, showing vascular dissection and tumor thrombus management.

Following the favorable response, the patient underwent cytoreductive surgery in March 2025. The operation was performed through an open midline laparotomy to allow for a rigorous, systematic, and complete abdominal exploration of all compartments, strictly adhering to the standard principles of peritoneal surface malignancy surgery. This thorough inspection included a complete omentectomy and targeted clearing of all macroscopically detectable lesions. Complete macroscopic resection of all visible metastatic disease was achieved, yielding a Completeness of Cytoreduction score of CC‐0, including excision of peritoneal implants (Figure 2A,B), segmental hepatectomy (Segments II and V) (Figure 2C), splenectomy, and thrombectomy of the infrahepatic inferior vena cava (Figure 2D). Additionally, a formal retroperitoneal lymph node dissection was executed, successfully clearing the previously documented regional nodal disease.

The postoperative course was complicated only by a paralytic ileus, which was successfully managed with conservative medical treatment and the patient was discharged after 7 days from the surgery. Postoperative histopathology revealed extensive tumor necrosis with < 1% residual viable tumor, limited to hepatic segment II. No viable tumor cells were identified in peritoneal, splenic, lymph node, or vascular margin samples, confirming R0 negative margins. The consensus conclusion was that the near‐complete tumor regression observed was predominantly attributable to the efficacy of the systemic therapy.

Systemic therapy was reinitiated 3 weeks after hospital discharge, maintaining the exact same preoperative drugs and scheduling (pembrolizumab 200 mg IV every 3 weeks and lenvatinib 20 mg orally daily). At the time of this report, the patient has completed 14 months of postoperative treatment and remains entirely asymptomatic with no evidence of disease recurrence. He continues under a strict surveillance protocol at our institution, consisting of physical examinations and laboratory evaluations on a monthly basis, alongside contrast‐enhanced imaging studies performed every 3 months.

## 3. Discussion

Peritoneal metastases are an exceptionally rare manifestation during the follow‐up of patients diagnosed with ccRCC. Several studies have estimated their incidence to be less than 1% in both clinical evaluations and autopsy series [2, 3], underscoring the unusual nature of our case. Crucially, our patient presented not with isolated peritoneal disease, but with a highly aggressive multicompartment recurrence involving the peritoneum, retroperitoneal lymph nodes, the venous system, and the liver parenchymal tissue. This simultaneous multifocal involvement represents a distinct, high‐volume metastatic scenario that carries an even worse prognosis than restricted peritoneal dissemination. Moreover, such metastases may arise even after complete surgical resection of the primary tumor, suggesting alternative dissemination pathways or delayed peritoneal seeding [4].

In recent years, the therapeutic landscape of metastatic ccRCC has been markedly transformed by immune checkpoint inhibitors, particularly the combination of pembrolizumab and lenvatinib. This regimen has demonstrated notable improvements in progression‐free survival, especially in patients with intermediate or favorable risk profiles. The pivotal CLEAR trial reported complete radiographic response rates of 18%–20% when this combination was used as first‐line therapy. However, these outcomes were primarily assessed using conventional imaging modalities, with comprehensive pathological evaluations rarely incorporated into the endpoints [5].

In contrast, our case contributes unique clinical and pathological evidence of a near‐complete response to immunotherapy. The histologic findings of extensive tumor necrosis and < 1% residual viable tumor are particularly striking. This result not only reinforces the efficacy of this treatment regimen but also prompts a reassessment of the predictive value of traditional radiologic criteria in metastatic RCC. The discordance between radiological and pathological findings observed here suggests that imaging may underestimate the true extent of tumor regression.

The concept of sequential treatment—systemic therapy followed by surgery—has gained increasing support. Several studies have evaluated deferred cytoreductive nephrectomy in patients receiving nivolumab‐based immunotherapy, either alone or with ipilimumab [6, 7]. Although these studies did not include pembrolizumab and lenvatinib, they collectively support the feasibility and potential benefit of surgery after a favorable systemic response. Our case further illustrates the viability of this strategy using a distinct therapeutic combination.

Additional support for this approach comes from recent case reports describing metastatic RCC patients treated with pembrolizumab and lenvatinib followed by cytoreductive surgery. In one report, a partial pathological response was observed with approximately 30% viable tumor remaining [8]; in another, a complete pathological response was documented [9]. Together with our experience, these cases highlight the potential for significant tumor regression and advocate for the judicious use of surgery in selected patients demonstrating robust systemic responses.

Despite these encouraging examples, cases like ours remain rare. Earlier reports of extensive peritoneal dissemination in RCC describe poor outcomes, particularly when patients present with ascites and carcinomatosis without prior surgery [10]. Other cases describe malignant ascites as the sole initial presentation, managed with immunotherapy alone [3]. In yet others, peritoneal progression occurred after nephrectomy without systemic therapy, with fatal outcomes [4].

The role of metastasectomy as part of an aggressive multimodal strategy in metastatic ccRCC remains debated. Retrospective analyses have identified several factors associated with poor cancer‐specific survival in patients undergoing cytoreductive surgery without prior systemic therapy—namely, advanced tumor stage, high histologic grade, nonpulmonary and multiorgan metastases, positive margins, and metastasis within 12 months of diagnosis [11]. Patients with four or more of these factors had notably poor outcomes, a risk profile that closely resembled that of our patient.

To successfully execute this strategy in a multicompartment setting, we adhered strictly to surgical oncological principles detailed in peritoneal surface malignancy literature, ensuring a CC‐0 status by systematically clearing the peritoneal implants, performing an omentectomy, and performing a formal retroperitoneal lymphadenectomy to clear the regional nodal disease. Nevertheless, more recent studies suggest favorable overall survival in patients who underwent complete metastasectomy following targeted therapy, even in cases initially deemed inoperable [12, 13]. Importantly, to our knowledge, no studies have specifically evaluated the combination of immunotherapy and tyrosine kinase inhibitors prior to metastasectomy with the aim of achieving complete macroscopic resection.

In this context, our case is singular. It documents, to the best of our knowledge, the first reported instance of ccRCC with multicompartment recurrence including peritoneal carcinomatosis treated with pembrolizumab plus lenvatinib followed by complete cytoreductive surgery, achieving a near‐complete pathological response across multiple anatomical compartments. Unlike prior reports where patients succumbed to the disease or were ineligible for such combined approaches, this case offers a compelling demonstration of the potential synergy between systemic immunotherapy and surgical intervention across multiple anatomic boundaries.

Collectively, this case supports the hypothesis that modern immunotherapy can fundamentally alter the natural history of metastatic ccRCC. Although prospective studies are warranted, deferred cytoreductive surgery following a strong systemic response may not only provide radiographic and clinical control but also offer a meaningful survival advantage in well‐selected patients with multicompartment disease.

## 4. Conclusion

Peritoneal metastases originating from ccRCC remain an unusual and often ominous clinical presentation, traditionally associated with limited therapeutic options and poor prognosis. This case, however, documents a highly atypical clinical trajectory in which upfront systemic therapy with pembrolizumab and lenvatinib enabled complete cytoreductive surgery and resulted in near‐complete pathological response of a multicompartment recurrence.

Beyond its rarity, this case illustrates how a staggered therapeutic approach, grounded in rigorous multidisciplinary assessment and strict surgical principles, can convert initially unresectable disease into an opportunity for complete surgical resection and durable disease control. Although similar strategies have been explored with other immunotherapy regimens, this is, to our knowledge, the first report to demonstrate such a profound pathological response using this specific combination in a multicompartment setting.

Ultimately, this case highlights not only the evolving role of systemic therapy in metastatic ccRCC but also the critical importance of individualized surgical planning and integrated oncologic care.

## Funding

No funding was received for this manuscript.

## Ethics Statement

Written informed consent was obtained from the patient for publication of this case report and any accompanying images. All procedures performed were in accordance with institutional guidelines and the Declaration of Helsinki.

## Conflicts of Interest

The authors declare no conflicts of interest.

## Supporting information


**Supporting Information** Additional supporting information can be found online in the Supporting Information section. Supporting Information. CARE (CAse REport) checklist (2013), completed for the present case report.

## Data Availability

The data that support the findings of this study are available on request from the corresponding author. The data are not publicly available due to privacy or ethical restrictions.
