# Supplementary material for: Achieving Complete Cytoreduction After Systemic Therapy for Stage IV Renal Carcinoma With Multicompartment Recurrence and Peritoneal Carcinomatosis: A Case Report
Source: Case Rep Oncol Med. 2026 Aug 27;2026:9036511. doi: 10.1155/crom/9036511 (PMC13519954; doi:10.1155/crom/9036511)
Supplement: Supplementary file 1 [file CROM-2026-9036511-s001.pdf]

CARE case report guidelines

CARE Checklist of information to include when writing a case report

(cc) BY-NC-ND

Topic	Item	Checklist item description	Reported on Line
Title	1	The diagnosis or intervention of primary focus followed by the words "case report"	Title page
Key Words	2	2 to 5 key words that identify diagnoses or interventions in this case report, including "case report"	Page with abstract (Key Words section)
Abstract (no references)	3a	Introduction: What is unique about this case and what does it add to the scientific literature?	Abstract paragraph, sentences 1–3
	3b	Main symptoms and/or important clinical findings	Abstract paragraph, description of initial presentation and metastatic progression
	3c	The main diagnoses, therapeutic interventions, and outcomes	Abstract paragraph, systemic therapy and surgical intervention description
	3d	Conclusion—What is the main "take-away" lesson(s) from this case?	Final sentence of abstract
Introduction	4	One or two paragraphs summarizing why this case is unique (**may include references**)	Introduction section
Patient Information	5a	De-identified patient specific information.	Case Presentation section (31-year-old male, de-identified)
	5b	Primary concerns and symptoms of the patient.	Case Presentation – initial flank pain and subsequent abdominal distension
	5c	Medical, family, and psycho-social history including relevant genetic information	Case Presentation – previously healthy, no relevant medical or family history reported
	5d	Relevant past interventions with outcomes	Case Presentation – radical nephrectomy (January 2023)
Clinical Findings	6	Describe significant physical examination (PE) and important clinical findings.	Case Presentation – imaging findings and histopathological results
Timeline	7	Historical and current information from this episode of care organized as a timeline	Chronological narrative provided in Case Presentation (January 2023–March 2025)
Diagnostic Assessment	8a	Diagnostic testing (such as PE, laboratory testing, imaging, surveys).	Case Presentation – CT, MRI, histopathology, staging, IMDC classification
	8b	Diagnostic challenges (such as access to testing, financial, or cultural)	No significant diagnostic challenges reported
	8c	Diagnosis (including other diagnoses considered)	Case Presentation – ccRCC pT3aN0M0; later metastatic ccRCC (Stage IV, M1)
	8d	Prognosis (such as staging in oncology) where applicable	Case Presentation – IMDC poor risk classification
Therapeutic Intervention	9a	Types of therapeutic intervention (such as pharmacologic, surgical, preventive, self-care)	Case Presentation – pembrolizumab plus lenvatinib and cytoreductive surgery
	9b	Administration of therapeutic intervention (such as dosage, strength, duration)	Case Presentation – pembrolizumab 200 mg IV every 3 weeks; lenvatinib 20 mg daily (14 cycles)
	9c	Changes in therapeutic intervention (with rationale)	Case Presentation – no dose modifications required
Follow-up and Outcomes	10a	Clinician and patient-assessed outcomes (if available)	Case Presentation – radiologic partial response (RECIST v1.1) and near-complete pathological response
	10b	Important follow-up diagnostic and other test results	Case Presentation – postoperative histopathology and ongoing surveillance
	10c	Intervention adherence and tolerability (How was this assessed?)	Case Presentation – treatment well tolerated without dose reductions
	10d	Adverse and unanticipated events	Case Presentation – grade 2 fatigue, grade 1 diarrhea, grade 2 hypertension
Discussion	11a	A scientific discussion of the strengths AND limitations associated with this case report	Discussion section – strengths and limitations addressed
	11b	Discussion of the relevant medical literature **with references**.	Discussion section with references 1–13
	11c	The scientific rationale for any conclusions (including assessment of possible causes)	Discussion section – rationale for immunotherapy and deferred cytoreduction
	11d	The primary "take-away" lessons of this case report (without references) in a one paragraph conclusion	Conclusion section
Patient Perspective	12	The patient should share their perspective in one to two paragraphs on the treatment(s) they received	Patient perspective not available
Informed Consent	13	Did the patient give informed consent? Please provide if requested	Yes ☒ No ☐
